# The safety and efficacy of fecal microbiota transplantation in a population with bipolar disorder during depressive episodes: study protocol for a pilot randomized controlled trial

**DOI:** 10.1186/s40814-021-00882-4

**Published:** 2021-07-14

**Authors:** Noah C. A. Cooke, Asem Bala, Johane P. Allard, Susy Hota, Susan Poutanen, Valerie H. Taylor

**Affiliations:** 1 Snyder Institute for Chronic Diseases, Cumming School of Medicine, University of Calgary, Calgary, Alberta Canada; 2 Department of Psychiatry, Cumming School of Medicine, University of Calgary, Calgary, Alberta Canada; 3 Department of Medicine, Toronto General Hospital, University of Toronto, Toronto, Ontario Canada; 4 Infection Prevention and Control Department, Department of Medicine, University of Toronto, Toronto, Ontario Canada; 5 Departments of Microbiology and Medicine, University Health Network and Sinai Health, University of Toronto, Toronto, Canada; 6 Hotchkiss Brain Institute, Cumming School of Medicine, University of Calgary, Calgary, Alberta Canada

**Keywords:** FMT, Bipolar disorder, Microbiome, Mood

## Abstract

**Background:**

Bipolar disorder (BD) is a chronic, debilitating illness with significant medical morbidity, often secondary to current treatments, and a high recurrence rate. This burden of disease reflects limitations in the tolerability and efficacy of current treatments. There is a compelling body of evidence linking the gut microbiota to mental illness, and while microbial manipulation via probiotic use has been studied as a therapeutic in BD, targeted trials of fecal microbiota transplantation (FMT) have not been conducted in this population.

**Methods and design:**

We describe a pilot randomized controlled trial of FMT in participants with BD depression to assess the feasibility, efficacy, safety, and tolerability of this intervention. Individuals between 18 and 65 years of age will be enrolled in the study if they meet diagnostic criteria for a major depressive episode of at least moderate severity in the context of a BD diagnosis and have not responded to treatment for BD. Participants will be randomized 1:1 to receive either screened and processed donor stool (allogenic FMT) or their own stool (autologous FMT) via colonoscopy and monitored for 24 weeks post intervention. Depressive and manic symptoms, treatment acceptability, and gastrointestinal and other side effects are assessed at baseline (prior to randomization) and weekly. Stool samples to assess microbiome composition are obtained at baseline and 3 and 6 months.

**Discussion:**

Currently, FMT represents a novel therapeutic option for treating BD depression. This protocol allows for the assessment of the feasibility, efficacy, acceptability, and safety of an intervention aimed at changing the microbiome in those with BD. Results from this pilot study will guide the development of larger trials of FMT for BD depression and may give more insight into how the gut microbiome are altered in those with BD depression.

**Trial registration:**

Clinical Trials Gov NCT03279224

## Background

Bipolar disorder (BD) is a chronic and debilitating condition with an estimated lifetime prevalence of over 2.0% [1]. While the illness is characterized in part by recurring episodes of mania or hypomania, depression accounts for up to 72% of time during which a person is ill [2]. The depressive components of the disorder are also especially challenging to treat and account for clinically significant residual morbidity [3]. A recent review of treatments found no high or moderate strength of evidence for any treatment of the depressive phase of this BD depression [4], supporting research into alternative therapeutic options.

Mounting evidence suggests that the MGB-axis, comprised of neural, humoral, and cellular routes connecting the gut microbiota with the central nervous system (CNS), is perturbed in neuropsychiatric disorders [5]. Essentially, the gut microbiota is able to modulate the MGB-axis both directly and indirectly via endocrine, neural, and immune pathways. In disease or stress states, these pathways may become dysregulated, resulting in intestinal dysbiosis, changes in mood, behavior, and cognition, and altered inflammatory levels [6]. Specific alterations in gut microbiome composition have been identified in individuals with BD [7–10] *and correlate with illness severity* [11]*.* These findings provide a compelling rationale to investigate whether microbiota-targeted interventions have potential for the treatment of BD.

Several strategies for restoring the MGB-axis to a healthy state have been explored in the treatment of psychiatric disorders. As opposed to short-term effects on the microbiome via probiotic [12] and antibiotic administration [13], it is the use of fecal microbiota transplantation (FMT) that may offer the best biotherapeutic option. It increases microbial diversity and does not result in microbiota dysbiosis as does antibiotic treatment. Unlike probiotics, whose colonization appears to be transient, FMT also affords long-term engraftment of the donor strains [14].

To date, no clinical trials have investigated the feasibility and efficacy of FMT in individuals with BD, but a recent systematic review of preclinical and clinical work reported a decrease in depressive and anxiety-like symptoms and behaviours resulting from the transplantation of healthy microbiota, providing support for this area of research [15]. Herein, we present the methodology of a pilot randomized controlled study that evaluates the feasibility of a trial protocol for FMT in participants with BD depression.

The primary objective of this protocol is to evaluate the feasibility of FMT in a BD population and to also examine FMT efficacy in reducing depression severity in BD patients on stable, first-line treatment for BD depression with a prospective, double-blind randomized controlled trial (RCT). This will guide the development of a larger multisite RCT and further our understanding of the potential biotherapeutic potential of FMT. Other secondary objective of the pilot study are to determine the acceptability, safety, and tolerability of FMT in this population.

## Methods

### Trial design

**Fig. 1:**
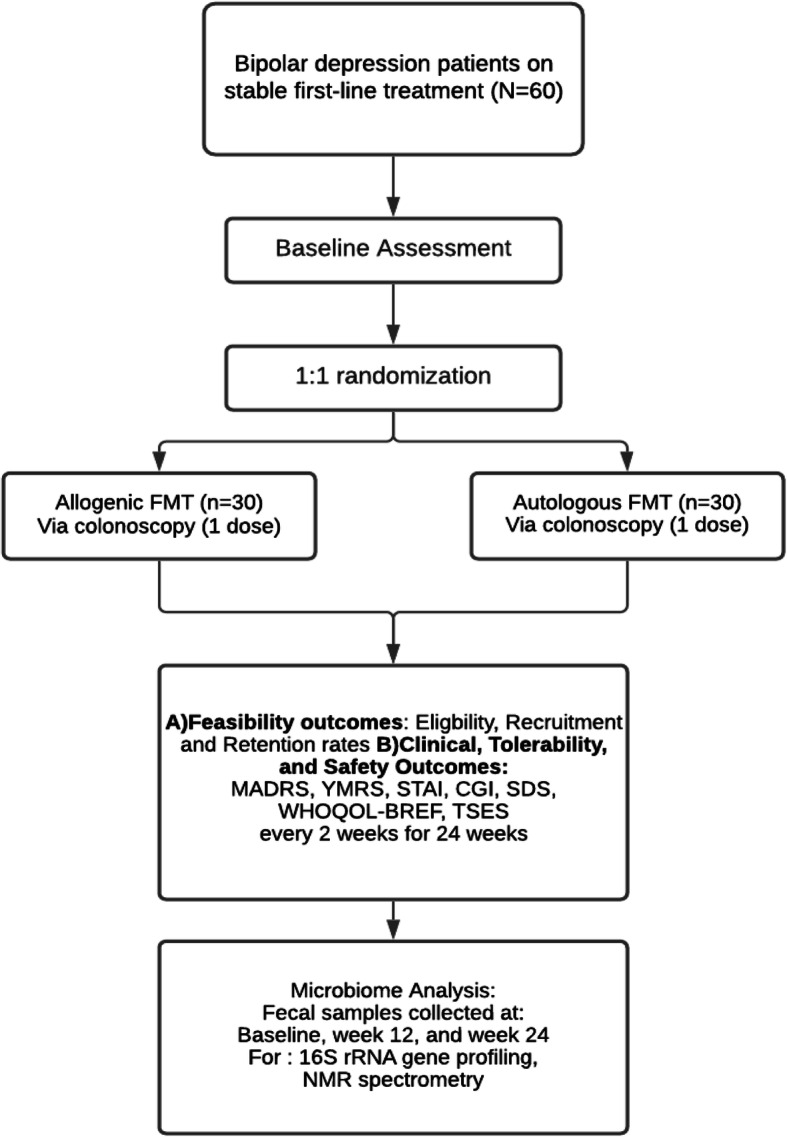
Trial design. Randomization schema of a pilot randomized controlled trial on the feasibility, safety and efficacy of FMT for Bipolar Disorder. *FMT*, fecal microbiota transplant; *MADRS*, Montgomery–Asberg Depression Rating Scale; *YMRS*, Young Mania Rating Scale; *STAI*, State Trait Anxiety Index; *CGI*, Clinical Global Impressions; *SDS*, Sheehan Disability Scale; *WHOQOL*, World Health Organization Quality of Life; *TSES*, Toronto Side Effect Scale

This study is a phase 2/3, double-blind, placebo-controlled RCT (See Fig. 1). Patients with BD on stable, first-line treatment will be randomized to receive either (1) allogenic FMT from a healthy donor or (2) autologous FMT with their own feces. After screening, FMT will be manufactured into an enema and administered via colonoscopy by a trained gastroenterologist. Participants will then be followed for 24 weeks for assessment of primary and secondary study outcomes. We plan to recruit 60 patients: 30 each for the intervention and control groups. This protocol is reported as per the SPIRIT guidelines [16].

The study will be conducted at the Psychiatry Department at Women’s College Hospital (Research Ethics Board [REB] # 2017-0099-B), with FMT preparation occurring in the University Health Network/Sinai Health System Department of Microbiology (REB# 17-0289-E) and FMT administration occurring at the endoscopy unit at University Health Network, Toronto Western Hospital (REB#17-5147). All hospital sites are affiliated with the University of Toronto. The study received a No Objection Letter from Health Canada (HC6-0240C201262). To help achieve adequate participant enrolment, flyers have been placed at the recruitment sites, and physicians providing clinical care at these sites have been informed of the study to assist with identification of potentially eligible participants. Physician education about the study has included small and large group presentations, as well as an information sheet.

The active phase of the study involves administration of 1 allogenic or autologous FMT course. Participants are then followed for 24 weeks, with stool samples taken and stored at baseline and 3 and 6 months. All study questionnaires will also be administered at each visit as well as review of side effects experienced. Outcome data collectors, participants, and physicians providing clinical care are blind to study group allocation.

The study is registered with ClinicalTrials.Gov under the identifier NCT02116127.

### Eligibility criteria

Eligible participants must be outpatients between 18 and 65 years of age, have a diagnosis of BD (type I or II) according to the Mini International Neuropsychiatric Interview (MINI) [17], have been on a stable first-line treatment for BD depression as defined in the Canadian Network for Mood an Anxiety Treatment (CANMAT) 2018 guidelines [18] at an adequate dose for ≥ 8 weeks prior to study entry, and currently be suffering from a depressive episode, characterized by a Montgomery–Asberg Depression Rating Scale (MADRS) [19] score at screening and baseline of ≥ 20. Patients will be excluded if they have a Young Mania Rating Scale (YMRS) [20] score of ≥ 12 at screening; meet DSM-IV criteria [21] for substance abuse within the last 6 months or lifetime dependency; have an active eating disorder; have schizophrenia or schizoaffective disorder; have chronic gastrointestinal diseases; conditions causing immunosuppression; a significant bleeding disorder; a history of incomplete colonoscopy; are pregnant or breastfeeding; have regularly used non-steroidal anti-inflammatory drugs, antibiotics, or iron supplements within 3 months prior to study entry; have used prebiotics or probiotics for medical purposes, or have taken antibiotics or any experimental drug within 3 months prior to study entry.

The healthy donors who provided the allogenic FMT will be recruited as part of the Microbial Therapeutics Outcome Program (MTOP) at University Health Network, Sinai Health System and University of Toronto. Potential donors will be excluded if they have a psychiatric disorder according to the MINI [17], a family history in first-degree (blood) relatives of psychiatric illness, a personal or family history of autoimmune disease in first-degree (blood) relatives; anaphylactic food or environmental allergies; diabetes or “pre-diabetes”, defined as HbA1c > 6%; are a smoker (cigarette, marijuana, other); use recreational drugs; regularly consume 2 or more alcoholic beverages daily; and regularly use iron supplements in the 3 months prior to study entry; have a history or symptoms suggestive of underlying gastrointestinal disease; infection or colonization with transmissible agents for repeated donation; active malignancy, or any cancer within the last 5 years, excluding basal cell carcinoma of the skin; risk factors for prion-related disease, including family history of Creutzfeld-Jacob Disease, corneal or dural transplant, or receipt of human-derived pituitary growth factor; history or high-risk status for recent acquisition of HIV, Hepatitis B, or Hepatitis C or sexually transmitted illnesses; immunosuppression; history or physical findings of chronic liver disease or cholestasis; evidence of active encephalitis or meningitis; evidence of active systemic viral, bacterial or fungal infection; receipt of live vaccine in preceding 30 days; receipt of blood transfusion from a country other than Canada in preceding 6 months; history of dementia or degenerative neurological disorders of unknown etiology; recent bite from an animal that may have rabies within the past 6 months; antibiotic use in the 6 months preceding donation; probiotic use for medicinal purposes in the 3 months prior to donation; use of cholestyramine within 3 months of donation; known and current history of blood in stools; and travel outside Canada or the USA in the past 6 months.

To ensure that recipients with severe or anaphylactic reactions to specific foods do not receive FMT from a donor who has recently consumed those foods, dietary screening utilizing a questionnaire developed by the MTOP program will be performed along with the regular prospective screening for medical health, travel and other relevant exposures using a brief checklist for consumption of common allergens. Detailed medical assessment will also be conducted to ensure potential donors are at low risk of infectious disease. This will be further confirmed by stool analysis, free from infectious agents.

### Recruitment

Potentially eligible participants who are willing to hear more about the study receive a detailed study explanation from the study research coordinator. Subjects are provided with a clear explanation of the objectives, procedures, risks, and benefits of the study, and all questions are answered. Questions are asked of subjects to ensure that they understand the nature of the research, risks, and potential benefits of study participation, and their rights as research subjects prior to their signing the informed consent document. The study coordinator will obtain informed, written consent during *Visit 1,* at which time further screening will be performed. The same qualified team members will enroll participants and stool donors. These individuals will not be involved in the clinical care of the patients. To maximize retention, patients will receive reminders of appointments via phone. If a patient misses an appointment, the reason will be investigated, and two attempts will be made to reach them by phone. Participants are given meaningful opportunities during the study to provide ongoing consent for continuation within the study protocol.

### Allocation of interventions

After informed consent is obtained, participants will be randomized to intervention and placebo control groups 1:1, according to a blocked randomization list (4 patients per block), generated in SAS proc PLAN by an individual at the University Health Network not involved in conducting or analyzing the study. Both the participant and study team, except for the lab technologist, will be blinded to group interventions. The allocation list will be kept in sequentially numbered envelopes with the group assignments. The lab technologist will maintain allocation concealment and prepare fecal filtrates for each patient but not be involved in any other part of the study. The researcher performing FMT will receive the designated fecal filtrate labeled only with the participants’ numbers. If an adverse event occurs that is thought to be potentially linked to FMT and requires immediate investigation, the code will be broken only for the affected patient. Only the study physician with the applicable expertise will be unblinded; the rest of the study team will not be informed.

### Intervention

Each participant receives one dose of either autologous or allogenic FMT product. FMT manufacturing will be performed as in a previous RCT (NCT01226992). Briefly, 50 g of screened donor or participant feces will be homogenized for 30 min using a Stomacher® Paddle Blender with 30 mL sterile 0.9 % NaCl + 10% glycerol in a sterile, 330-μn microfilter-separated, double-compartment polyethylene bag. The autologous FMT control arm is essential because bowel preparation and colonoscopy can induce changes to the intestinal microbiome [22, 23]. The volume of fecal filtrate corresponding to 50 g of original feces will be aliquoted, labeled with a recipient ID number to maintain blinding, and stored at – 80 °C. Freezing stool with glycerol as a cryoprotectant has been shown to yield active FMT preparations [24, 25]. For instance, frozen fecal filtrate for treatment of *Clostridium difficile* infection can be stored for at least 6 months in 10% glycerol without loss of clinical efficacy or viability of the six bacterial groups measured [26]. Similarly, clinical efficacy in treating *C. difficile* infection was shown not to differ between frozen and lyophilized FMT product after 9, 11, or 15 months [27]. An aliquot of the final fecal filtrate will be stored at – 80 °C for analysis in case retroactive testing of the fecal filtrate is required.

#### FMT delivery and mucosal biopsies

Prior to colonoscopy, three falcon tubes of frozen fecal filtrate, corresponding to a total of 150 g original donor feces, will be thawed at room temperature over 2.5 h. Thawed feces will be diluted to a total volume of 300 mL with 0.9 N NaCl and packaged into 6 × 50 mL syringes for colonoscopy. The evening prior to colonoscopy, study subjects will take 4L of polyethylene glycol laxative solution. Colonoscopy will be performed by an experienced gastroenterologist at the University Health Network. First, six mucosal pinch biopsies (3 mm size) will be taken: two each from the end of the rectum (20 cm from the anal verge), the ascending colon, and the terminal ileum. These samples will be used for compositional and metabolomic profiling of the mucosa-adherent microbiome. Biopsies will also be sent to pathology if clinically needed. Second, the gastroenterologist will collect 5 mL of colon secretion into sterile microtubes (secretions will be collected via trap). This will be frozen immediately and stored at – 80 °C and used to test for pathogens at the time of FMT in case a recipient develops an infectious disease. Finally, participants will be placed in the right lateral recumbency position, and FMT will be performed with fecal filtrate in one syringe deposited in the terminal ileum and five in the cecum.

### Data collection, timeline, and outcomes

#### Outcomes

The study visit schedule is detailed in Table 1. A description of the tools used to measure specific outcomes is provided below. The primary outcome measures for the pilot study are (1) feasibility, (2) acceptability, and (3) adherence with the trial protocol.

**Table 1:** Study schedule for the participants in the pilot randomized controlled trial of fecal microbiota transplantation (FMT) treatment of bipolar depression

Measure	Study period					Purpose
	Prior to intervention		Intervention	Follow-up (12 visits every 2 weeks)		
	Screening	Baseline	Colonoscopy visit	Every 2 weeks	Week 12 and week 24 visits	
Eligibility screening	**X**					**Feasibility**
Informed consent	**X**					**Feasibility**
Confirmation of eligibility		**X**				**Feasibility**
Randomization		**X**				**Feasibility**
Colonoscopy			**X**			**Intervention**
Anthropometric measurements		**X**		**X**		**Covariates**
Food diary		**X**			**X**	**Covariates**
Mucosa biopsies/colon secretion			**X**			**Microbial analysis**
Stool sample		**X**			**X**	**Microbial analysis**
Blood sample			**X**			**Safety**
Concomitant medications	**X**	**X**	**X**	**X**		**Safety**
Symptoms diary				**X**		**Safety/tolerance**
Toronto Side Effect Scale (TSES)		**X**		**X**		**Safety/efficacy**
Mini-International Neuropsychiatric Interview (MINI)	**X**					**Feasibility**
Young Mania Rating Scale (YMRS)	**X**	**X**		**X**		**Efficacy**
Montgomery–Asberg Depression Rating Scale (MADRS)	**X**	**X**		**X**		**Efficacy**
State and Trait Anxiety Inventory (STAI)		**X**		**X**		**Efficacy**
Sheehan Disability Scale (SDS)		**X**		**X**		**Efficacy**
World Health Organization Quality of Life (WHOQOL-BREF)		**X**		**X**		**Efficacy**

##### Feasibility, clinical, tolerability, and safety outcomes

We will record feasibility data related to (i) eligibility (for example, proportion of mental health clinic patients eligible), (ii) recruitment (for example, number, nonparticipation reasons, especially the decision related to the colonoscopy aspect of the study), and (iii) timing (for example, time before participant begins treatment).

Safety of FMT will be assessed from unsolicited and solicited adverse events. Clinical and tolerability scores at baseline (Visit 2) will be compared with those at study completion (Visit 15). In addition, the Toronto Side Effects Scale (TSES) [28], a 32-item instrument designed to establish incidence, frequency, and severity of CNS, gastrointestinal, and sexual side effects will be used.

##### Assessment tools

The following outcome measures will be used to assess symptoms: the MADRS [19], a 10-item questionnaire which psychiatrists use to measure the severity of depressive episodes in patients with mood disorders; the YMRS [20], which is 11 items and is a clinician-administered interview scale used to assess manic symptoms; the State Trait Anxiety Index (STAI) [29], a commonly used measure of anxiety that can be used to diagnose anxiety and to distinguish it from depressive syndromes; the Clinical Global Impressions (CGI) rating scales [30], commonly used to measure symptom severity and treatment response in studies of patients with mental disorders; and the Sheehan Disability Scale (SDS) [31], a brief self-report tool developed to assess functional impairment in work/school, social life, and home life or family responsibilities domains. The World Health Organization Brief Quality of Life Scale (WHOQOL-BREF) [32], a quality of life assessment designed to be applicable cross-culturally, will also be administered.

##### Microbiome composition

Fecal microbiome profiling will be performed using Illumina sequencing. DNA extraction will be performed using a previously described protocol that enhances DNA recovery from microbial communities [33, 34], with modifications [35]. Total eubacterial load will be measured via Quantitative polymerase chain reaction (qPCR) with universal primers 8fM and Bact515R [36]. Thermal cycle reactions will run on the Bio-Rad CFX96 Real-Time System. Samples will be prepared in triplicates and quantified based on a standard curve with an efficiency of 94.9% generated by 10-fold serial dilutions of a plasmid containing a single copy of the 16S rRNA gene. The negative control includes a no template reaction to control for reagent contamination.

Bacterial community profiling of the 16S rRNA gene will be performed using paired-end reads of the V3 region using bar-coded Illumina sequencing as described previously [37] with bar codes included in the forward primer. Alternatively, amplification of the V3–V4 region will be performed using 806R primer [38]. Amplified product will be purified by separation via agarose gel electrophoresis and gel extraction. Pair-end sequencing will be performed on a MiSeq Illumina sequencer as per manufacturer’s instructions, as described previously [39].

##### Microbiome metabolomics

Fecal samples will be collected and stored at − 80°C, and fecal supernatants will be analyzed via NMR spectrometry [40].

##### Mucosal biopsy analysis

Of the two biopsies from each of the three locations, one will be stored in 10% formalin for pathological assessment and the other stored at – 80 °C for microbiome analysis. DNA will be extracted from 3 mg of tissue and purified as for the stool. Sequencing and analysis will be performed as for stool samples.

A baseline visit with the research coordinator (blind to group allocation) occurs in person, prior to the start of the active study phase, to collect baseline demographic and health service use information as well as baseline measures on mental health symptom scales. During all subsequent visits (visit 1 to visit 15), concomitant medications will be recorded. Anthropometric measurements (waist-to-hip ratio, and body mass index (BMI)) will be taken at every visit, except during the first visit for screening (visit 2—visit 15). Food intake will be recorded for 3 days (2 weekdays and 1 weekend day), and the completed forms will be collected during visit 2 (baseline), 9 (12 weeks), and 15 (24 weeks). To minimize confounding effects, participants will be asked to maintain their usual diet and exercise level. Participants will also itemize their food intake using a validated form developed by the Fred Hutchinson Cancer Research Center [41]. Food records will be readministered at 3- and 6-month time points. Dietary data will be analyzed using NUTRITIONIST PRO™ DIET ANALYSIS SOFTWARE (Axxya Systems) to assess intake of macro- and micronutrients.

##### Pre-intervention assessments

At *Visit 1*, informed written consent will be obtained, and demographic characteristics, current medical conditions and concurrent medications will be recorded. The following questionnaires will also be administered: MINI [17] for diagnosis of BD, YMRS [20] for mania, and MADRS for depression [19]. Recipients will receive instructions and two kits for collecting two stool samples at home, and a standard food record to measure dietary intake for 3 days. As diet and bowel preparation alter the microbiome, stool samples will be collected at *Visit* 2, 3 to 4 days prior to colonoscopy. A fraction of these fecal samples will be stored at – 80 °C for microbiome analysis. After randomization, 150 g of the remaining, refrigerated feces will be manufactured into FMT product. A portion of FMT fecal filtrate will be frozen at – 80 °C for future analysis, and 20mL of blood will also be collected prior to randomization for analysis if a recipient is later found to be infected with a transmissible disease. For baseline clinical scores, the following questionnaires will be given: MADRS [19], YMRS [20], CGI [30], STAI [42], SDS [31], WHOQOL-BREF [32], and the TSES [28].

##### Intervention visit

At *Visit 3*, FMT will be performed. To document side effects, patients will be provided with a symptom diary after FMT, which they will submit at every subsequent study visit.

##### Post-intervention visits

To evaluate whether FMT effects longer-term changes in depression severity and microbiome profile, patients will be followed for 6 months (24 weeks) post-FMT, during *Visits 3–15*. Clinical (MADRS, YMRS, CGI, STAI, SDS, WHOQOL-BREF) and tolerability (TSES) outcomes will be assessed every 2 weeks after FMT for 6 months. Adverse events will be verbally screened for biweekly. Stool samples will be collected at 3 and 6 months to assess fecal microbiome composition post-FMT.

### Statistical methods

We will use descriptive methods to estimate feasibility and compliance with the FMT intervention, the number of FMT and placebo treatments, the number of fecal samples provided, the duration of follow-up, recruitment rate, rates of nonparticipation, and so on. We will calculate acceptability using reported side effects and patient outcomes.

Variable distribution will be assessed using graphical methods and descriptive statistics, including means, standard deviations, medians and 1st and 3rd quartiles, frequencies and proportions, will be calculated where appropriate. Randomized groups will be compared to assess whether imbalance in covariates such as age and gender are present. For continuous measures (e.g., MADRS scores), change scores will be calculated as the difference between baseline and 6-month visit assessments. Groups will be compared using independent samples *t* test or Wilcoxon signed rank test as appropriate. For binary measures (e.g., proportion of side effects), the difference between groups will be tested using Chi-square or Fisher exact test as appropriate. Bivariate relationships between outcome measures and changes in microbiome composition (e.g., relative abundances of taxa of interest) will be explored using Spearman and Pearson correlation coefficients. For microbiome and metabolome data, multivariate dimensionality reduction approaches, such as Principal Components analysis and cluster analysis, will be employed.

Trajectory plots will be created to examine changes in outcome measures over time. Generalized linear regression models will then be used to assess differences [43] between groups at different time points, as was well as to explore the relationship between these changes and microbiome characteristics. In order to account for correlation between time points, random effects models and generalized estimating equations approach will be used. All models will include group, time, and group by time interaction terms; additionally, factors with known influence on outcome measures will be considered for inclusion as covariates, particularly if found imbalanced between the randomized groups. For patient dropout, the missingness patterns will be assessed, and appropriate methods of handling missing data will be used [44]. Goodness-of-fit will be applied for model diagnostics.

Statistical analyses will be performed using SAS 9.4, R 3.1, as well as analytic tools available in QIIME package. All tests will be two-sided with a significance level of 5%. Where necessary, *p* value adjustments for multiple testing (e.g., Bonferroni, Holm’s Bonferroni Step-down, Benjamini and Hochberg False Discovery Rate) will be employed.

### Sample size

This is a pilot feasibility trial designed to show if a larger RCT trial would be worthwhile. Therefore, no formal sample size calculation has been carried out [45]. A review by Hertzog suggests a range of 20 to 40 participants to allow for sufficient variability in acceptability assessment of an intervention [46]. Based on work from a pharmaceutical study in a similar patient population [47], the expected standard deviation of change expected in the MADRS score is approximately 12. Therefore, with 25 patients per group, we will be able to quantify the difference in change in MADRS score between groups to within 6.5 points with 95% confidence. This will allow data that can be used in the generation of a sample size for a larger trial. To account for a 15% dropout rate, we plan to recruit 60 patients; 30 will receive the active treatment (allogenic FMT), and 30 will receive the control treatment (autologous FMT).

### Data management and monitoring

#### Data monitoring and auditing

A Data Monitoring Committee will identify mechanisms to ensure the safety and efficacy of all aspects of the study. This committee will review the research protocol, informed consent documents, plans for data and safety monitoring, and steps taken to maintain patient data confidentiality. Furthermore, the committee will monitor all stages of the study, including factors that may influence study quality (data quality and timeliness, participant recruitment, accrual and retention, participant risk versus benefit); consider new scientific or therapeutic evidence with implications for the safety and ethicality of the study; and review serious adverse event documentation and safety reports. Adverse events will be assessed verbally. Additionally, patients will be provided with a symptom diary covering symptoms that may indicate an adverse event related to FMT in the 7 days following the intervention. A physician will be responsible for all trial-related medical decisions and review of adverse events and severe adverse events throughout the study. All unanticipated problems will be reported to the local and main REBs. Adverse drug reactions (adverse events related or potentially related to the study project that are both serious and unexpected) will be subject to expedited reporting to Health Canada.

### Dissemination

Results will be published in scientific journals and presented at research conferences. We will also share our results via the “Gut Microbiota for Health Experts exchange,” an online community where experts in microbiome and health-related fields share news, innovation, and information on topics pertaining to the gut microbiota. The results will also be made available to individuals suffering from BD, who are public stakeholders in the study, via advocacy groups.

## Discussion

Bipolar disorder is a common and disabling mental illness with significant morbidity and mortality. People living with BD experience substantial impairment, being symptomatic with syndromal or subsyndromal symptoms, particularly those of depression, for approximately half of their lives [48]. It is chronic and highly recurrent, with close to 60% of the patients experiencing a recurrence of BD in the first 2 years, and about 75% experiencing a recurrence in over 5 years following the initial diagnosis [49]. Reoccurrence often occurs in the face of treatment noncompliance, which is due in part to the significant side effects associated with BD treatments [50], but it can also occur when there is adherence to a treatment plan [51]. There is therefore a need to locate treatments with better tolerability and efficacy. Compelling evidence supports an MGB-axis link and as a recent review concluded, there is growing support for the treatment and transmission of psychiatric illnesses through FMT. Further research with stronger scientific design is warranted in order to fully determine the efficacy and safety of this potential treatment [15].

The strengths of our protocol include (1) the use of a novel treatment option for BD depression that has not been previously described in this population; (2) a strong interdisciplinary team of coinvestigators to ensure participant safety via the use of well screened and processed FMT; (3) extensive follow-up, to monitor depressive symptoms 6 months after the intervention; (4) study oversight by a strong team with a diverse range of expertise and training in the fields of neuroscience, gastroenterology, infectious disease, and microbiology; and (5) a comprehensive dissemination plan to ensure adequate uptake of the knowledge that this study will generate. There are some limitations to the pilot study methodology. First, we restricted our sample recruitment to those who failed previous treatments. Thus, the effect of FMT within our treatment-resistant population may not represent its effect in a nonresistant population. Second, we excluded those who had gastrointestinal issues such as IBD, conditions which could alter outcomes of FMT. The nature of the colonoscopy intervention may also result in particular patient selection, excluding those medically unable to tolerate this procedure. Finally, because this is a pilot study, our results will not support definitive conclusions on efficacy or safety of FMT in BD depression but may help guide the development of a larger study to focus on these outcomes.

In summary, this protocol was developed through strong collaborations between psychiatry, gastroenterology, and infectious disease delivery providers and research practices and represents a multisite hospital collaboration. This pilot RCT will allow us to assess the feasibility of novel treatments for BD depression and to understand potential mechanisms of how microbial manipulation can impact disease outcomes. This will help us guide the development of larger trials in this area.

### Study status

Participant enrollment began February 1st, 2017. We have currently enrolled 25 participants.

## Data Availability

The datasets used and/or analyzed during the current study are available from the corresponding author on reasonable request.

## Abbreviations

BD: Bipolar disorder
FMT: Fecal microbiota transplantation
MGB: Microbiota−gut−brain
CNS: Central nervous system
RCT: Randomized controlled trial
MINI: Mini International Neuropsychiatric Interview
MADRS: Montgomery–Asberg Depression Rating Scale
YMRS: Young Mania Rating Scale
MTOP: Microbial Therapeutics Outcome Program
STAI: The State Trait Anxiety Index
CGI: Clinical Global Impressions
SDS: Sheehan Disability Scale
WHOQOL-BREF: World Health Organization Brief Quality of Life Scale
qPCR: Quantitative polymerase chain reaction
NMR: Nuclear magnetic resonance
BMI: Body mass index
